# Cranial Neuropathy Secondary to Carotid Artery Dissection: Clinical Features and Long-Term Outcomes

**DOI:** 10.3390/jcm14196854

**Published:** 2025-09-27

**Authors:** Helena K. Xeros, Irem Yesiloglu, Zafer Keser

**Affiliations:** Department of Neurology, Mayo Clinic College of Medicine & Science, Rochester, MN 55905, USA

**Keywords:** cranial neuropathy, carotid artery dissection, compressive neuropathy

## Abstract

**(1) Background:** Cranial neuropathy is a commonly encountered condition with various underlying etiologies. While carotid artery dissection (CAD) is a well-recognized cause of ischemic stroke, CAD-related cranial neuropathy is rare and poorly characterized. We have conducted a comprehensive review of the published literature to better characterize its clinical course and outcomes. **(2) Methods:** We systematically reviewed the PubMed, CENTRAL, Ovid MEDLINE, and Embase literature for CAD-related cranial neuropathy. Data extracted included demographics, affected cranial nerves, symptoms, time course, diagnostic approach, and therapeutic interventions. **(3) Results:** From 635 screened studies, 97 met the inclusion criteria, yielding data on 108 patients with CAD- or dissecting pseudoaneurysm (dPSA)-related cranial neuropathy. The hypoglossal nerve (CN XII) was most commonly affected (76%), and the distal cervical internal carotid artery was the most frequently involved segment (89%). Most patients (90%) were treated with antithrombotic therapy which included either antiplatelets (47%) or anticoagulants (43%). Thirteen patients (12%) underwent endovascular intervention, nearly all with a diagnosed dPSA (mean size, 14.8 mm). Outcomes were favorable, with 94% experiencing symptom improvement. **(4) Conclusions:** Despite inherent limitations, our study demonstrates that CAD-related cranial neuropathy is typically a benign condition that has excellent outcomes with medical management. Endovascular treatment is rarely performed and is primarily reserved for cases involving diagnosed dPSA.

## 1. Introduction

Carotid artery dissection (CAD) is recognized as an uncommon but clinically relevant cause of ischemic phenomenon, particularly in younger adult populations [1,2]. Spontaneous CAD can occur in young, healthy individuals due to an underlying, often subclinical, connective tissue vulnerability that weakens the arterial wall, making them susceptible to dissection after minor triggers such as trivial trauma, infection, or physiological stress [3]. While ischemic phenomenon is the most well-recognized presentation, CAD exhibits a wide spectrum of clinical manifestations. These range from entirely asymptomatic cases to local symptoms such as neck pain or Horner syndrome, as well as compressive phenomena involving adjacent structures [4,5]. Among these clinical presentations, cranial neuropathy represents a relatively rare but clinically relevant manifestation, occurring in approximately 10–12% of CAD cases [6,7,8].

Cranial neuropathies often arise secondarily to mass effect from an expanding intramural hematoma or formation of dissecting pseudoaneurysm (dPSA) of the internal carotid artery (ICA) [6,8,9]. Other potential mechanisms for cranial neuropathies associated with carotid artery dissection, particularly involving nerves not anatomically adjacent to the carotid artery, include vascular compromise of ICA branches supplying the cranial nerve trunks, as well as persistent vascular anatomical variations such as embryonic carotid–basilar anastomotic vessels [7,10,11]. It is important to distinguish cranial neuropathies associated with vertebral artery dissection, which more commonly result from ischemic stroke in the brainstem rather than direct compressive effects [6,12]. The optimal diagnostic approach and management strategies for this subset of CAD clinical sequelae remain poorly characterized, likely due to its relative rarity and clinical heterogeneity.

This comprehensive review synthesizes the existing literature on patient cases of CAD-associated cranial neuropathy, with the goal of clarifying the clinical course, management strategies, and outcomes. Furthermore, this review identifies key gaps in current knowledge to inform future clinical research and improve therapeutic decision-making in this rare but impactful clinical presentation.

## 2. Methods

The protocol for this review has been registered in the Open Science Framework (OSF) (Washington, DC, USA) (Registration DOI: 10.17605/OSF.IO/BR5TZ).

### 2.1. Search Strategy

A comprehensive literature search of the English literature was conducted utilizing PubMed, CENTRAL, Ovid MEDLINE, and Embase platforms to identify relevant studies on CAD-related cranial neuropathy from inception to May 2025. We followed PRISMA guidelines, and search strategies were created using a combination of keywords and standardized index terms: “(cranial neuropathy OR cranial nerve palsy OR nerve palsy) AND (carotid artery dissection OR carotid dissection OR carotid artery aneurysm OR carotid aneurysm OR carotid artery pseudoaneurysm OR carotid pseudoaneurysm).” The specific search terms can be found in Supplementary Table S1.

Studies were eligible for inclusion if they reported on spontaneous or traumatic cervical or intracranial CAD or dPSA with associated cranial neuropathy. All study designs were eligible for inclusion if individual patient data was available. Exclusion criteria included cases in which cranial neuropathy was attributed to causes other than secondary to CAD or dPSA, absence of imaging or clinical follow-up beyond the initial presentation, and publications in the form of reviews, commentaries, or opinion pieces. Additional exclusion criteria included non-English-language articles, pediatric patients (age < 18 years), iatrogenic dissections, procedure-related cranial neuropathies, and cranial neuropathies resulting from ischemic phenomena in the brain.

### 2.2. Study Screening Process

All identified articles were imported into EndNote and Covidence for deduplication and screening. Two independent reviewers (HX, IY) performed the initial screen of titles and abstracts to assess eligibility based on pre-defined inclusion and exclusion criteria as described above. Disagreements were resolved by consensus by the two reviewers. If a consensus was not reached, the third reviewer (ZK) served as a final adjudicator. Articles that met the initial inclusion criteria underwent full-text screening performed by two independent reviewers (HX, IY) for final inclusion. Disagreements on full-text inclusion were resolved by consensus or consultation with a third reviewer (ZK).

### 2.3. Data Extraction and Outcome Measures

Data extraction was completed by the two independent reviewers (HX, IY), and a standardized form was developed for data extraction. Once the data collection form was finalized and formal data extraction was completed, disagreements were resolved by consensus or consultation with a third reviewer (ZK). In the event of uncertainty about missing or unclear study data, study authors were contacted for clarification. Extracted variables included bibliographic information, study design, sampling method, sample size, patient characteristics (age, gender, race, ethnicity), treatment characteristics (type, dose, frequency and duration), type of control group, type of function-related outcomes reported, and duration of clinical and radiographical follow-up.

### 2.4. Bias Assessment

Two independent reviewers (HX, IY) assessed the risk of bias of each study, using the Joanna Brigg Institute (JBI) Critical Appraisal Tool [13] to assess case reports and cases series. Disagreements were resolved by consensus or consultation with a third reviewer (ZK).

### 2.5. Statistical Analysis

Given the limited number of patient cases available and variability in reporting, only descriptive statistical analyses were conducted. Continuous variables are presented as means with range (mean (range)), and categorical variables are reported as frequencies (number (percentage)).

## 3. Results

### 3.1. Literature Search Results

**Figure 1:**
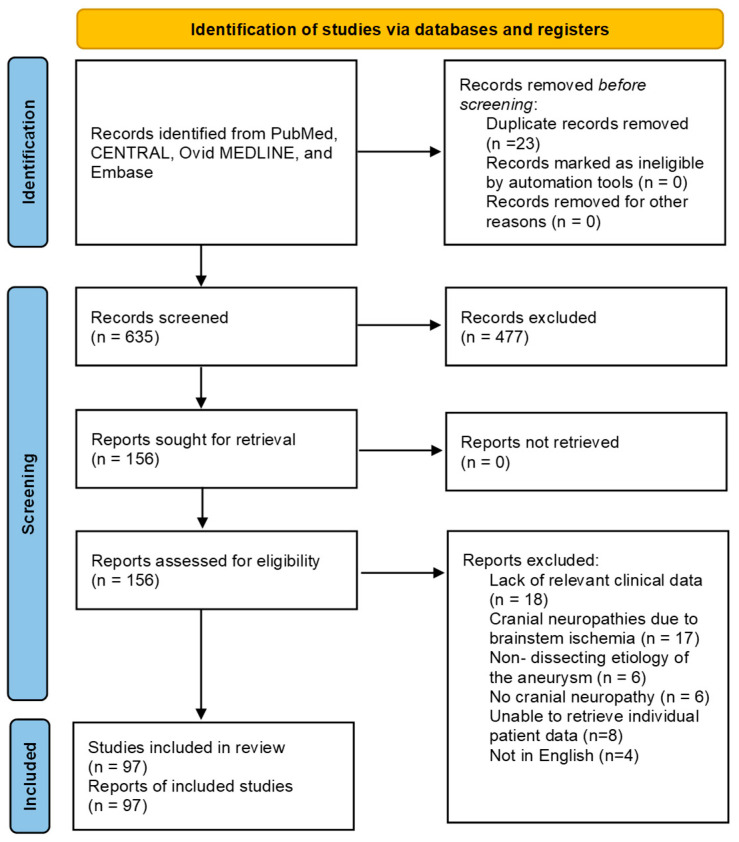
PRISMA flow diagram of study review process [106].

Following the literature search, 635 studies were identified and included for title and abstract screening. After irrelevant studies were excluded using title and abstract screening, 156 studies were retrieved for full-text screening. During the full-text screening, 59 studies were excluded due to a lack of relevant clinical data (n = 18); cranial neuropathy attributed to central ischemia (n = 17); no cranial neuropathy reported (n = 6); etiology inconsistent with dissection (n = 6); absence of individual patient data (n = 6); not being in English (n = 4); or inappropriate study design (n = 2) (Figure 1). A total of 97 studies were included for analysis with extractable data (Table 1) [8,9,10,11,12,14,15,16,17,18,19,20,21,22,23,24,25,26,27,28,29,30,31,32,33,34,35,36,37,38,39,40,41,42,43,44,45,46,47,48,49,50,51,52,53,54,55,56,57,58,59,60,61,62,63,64,65,66,67,68,69,70,71,72,73,74,75,76,77,78,79,80,81,82,83,84,85,86,87,88,89,90,91,92,93,94,95,96,97,98,99,100,101,102,103,104,105]. The individual baseline characteristics, antithrombotic treatments, interventions, and reported outcomes of the included studies are presented in Supplementary Table S2.

### 3.2. Risk-of-Bias Assessment

We performed bias assessment only with the Joanna Briggs Institute (JBI) Critical Appraisal Tool for case reports/case series, and we summarize this within Supplementary Table S3. Most studies had a low risk of bias.

### 3.3. Baseline Characteristics

From 97 studies, we identified a total of 108 patients (26 females) with cranial neuropathy secondary to CAD or dPSA (Table 1). The mean age was 48 years (26–68). Comorbidities included the following: twenty-two patients (20.37%) had hypertension, seven patients (6.48%) had migraines, six patients had a history of smoking (5.56%), four patients (3.70%) had a history of non-specific headaches, and two (1.85%) patients had hyperlipidemia. Other associated conditions included three patients (2.77%) with fibromuscular dysplasia (FMD), one (0.92%) with osteogenesis imperfecta, and one (0.92%) with Takayasu arteritis. In addition to involvement of the affected ICA segment, a subset of patients experienced additional dissections within other arteries: three patients (2.77%) had an additional vertebral artery dissection (no brainstem ischemia that would account for cranial neuropathy), two (1.85%) had a contralateral ICA dissection, one (0.92%) had recurrent ICA dissections, and one (0.92%) had middle cerebral artery dissection. The patients with FMD also had splenic, vertebral, and contralateral ICA dissection, and the patient with Takayasu arteritis had subclavian artery dissection. All reported cases of CAD or dPSA either occurred spontaneously or secondarily to minor trauma.

### 3.4. Clinical Course

The most frequently involved cranial nerve was the hypoglossal (CN XII), which was reported in 82 patients (75.92%), followed by the vagus (CN X) (n = 38, 35.18%) and the glossopharyngeal (CN XI) (n = 21, 19.44%). Lower cranial nerve involvement (defined as involvement of cranial nerves IX, X, XI, and XII) was present in 88 of 108 patients (81.48%). Of the 82 patients with CN XII involvement, 45 patients (54.87%) had isolated CN XII neuropathy, while 37 (45.12%) had additional cranial neuropathies, which included facial (CN VII) cranial neuropathy in three patients, vestibulocochlear (CN VIII) in one patient, and trigeminal (CN V) in one patient. Additionally, the upper cranial nerves, which include the oculomotor (CN III), trochlear (CN IV), trigeminal, and abducens (CN VI) nerves, were affected in 16 patients. Of these 16 patients, 11 cases were isolated, and 5 cases involved a combination of two or more of these nerves. Among patients with lower cranial nerve involvement, 66 cases (78.6%) were spontaneous and 18 (21.4%) were related to minor trauma. In those with upper cranial nerve involvement, 17 cases (85%) were spontaneous and 3 (15%) were related to minor trauma. Of the patients with combined upper and lower cranial nerve involvement (n = 4), 3 cases (75%) were spontaneous and 1 (25%) was related to minor trauma. In addition, there was no observed difference in upper versus lower cranial nerve involvement among patients with comorbidities such as fibromuscular dysplasia (FMD) or osteogenesis imperfecta; specifically, among patients with FMD, two presented with lower cranial nerve palsies and one with upper cranial nerve palsy, while the single patient with osteogenesis imperfecta exhibited lower cranial nerve involvement. The distal cervical ICA was the most frequently involved vascular segment, reported in 96 patients (88.88%), followed by the mid-cervical ICA (n = 23, 21.30%) (Table 1).

A total of 36 patients (33.3%) were diagnosed with pseudoaneurysm (dPSA). Patients with dPSA had lower cranial nerve involvement more frequently (n = 31, 86.1%), compared to those not diagnosed with dPSA (n = 57, 79.2%). Of patients with diagnosed dPSA, seven (19.4%) had CN VI, four (11.1%) had CN IV, three (8.3%) had CN III (8.3%), and three (8.3%) had CNVII involvement. Of patients without diagnosed dPSA, eight (11.1%) had CN V, six (8.3%) had CN III, four (5.6%) had CN VII, one (1.4%) had CN VIII, and one (1.4%) had CN VI involvement.

Out of 108 patients, 100 (92.59%) were clearly reported to have received antithrombotic therapy or were explicitly followed without medication (Table 2). Out of 100 patients, 47 (47%) received antiplatelet therapy (35 received single antiplatelet therapy (35%) and 12 received dual antiplatelet therapy (12%)). Among the 12 patients who received dual antiplatelet therapy, 10 were treated with aspirin and clopidogrel. For the remaining two patients on dual antiplatelet therapy, the agents used were not specified. Forty-three patients (43%) received anticoagulation. Oral anticoagulation (warfarin, phenprocoumon, or fluindion) was reported in 25 patients (25%), and parenteral heparin-based therapy at therapeutic doses was used in 10 patients (10%). For the remaining eight patients receiving anticoagulation (8%), no details were reported on the type of anticoagulation agent used. Of the total patient cohort with reported treatment decisions, 10 (10%) did not receive any antithrombotic therapy, which was clearly reported in the studies, and the reasons for withholding treatment were not specified.

In patients with a diagnosed dPSA, there were equal numbers that were treated with dual antiplatelet therapy (n = 8, 22.2%) and single antiplatelet therapy (n = 8, 22.2%). In contrast, patients without dPSA were more commonly treated with single antiplatelet therapy (n = 27, 37.5%) rather than dual antiplatelet therapy (n = 4, 5.6%). Anticoagulant therapy use was similar in both groups (n = 14, 38.9%; n = 29, 40.3%).

To evaluate the impact of the CADISS trial on treatment choices, patients were stratified based on publication year into pre-CADISS (prior to 2017) and post-CADISS (2017 and after). A total of 90 patients (90%) received any antithrombotic therapy, 63 (63%) were from pre-CADISS studies and 27 (27%) were from post-CADISS studies. Among the 63 pre-CADISS patients, 39 (61.90%) received anticoagulation, 20 (31.74%) received single antiplatelet therapy, and 4 (6.35%) received dual antiplatelet therapy. Among 27 post-CADISS patients, 4 (14.81%) received anticoagulation, 15 (55.56%) received single antiplatelet therapy, and 8 (29.62%) received dual antiplatelet therapy. The mean duration of antithrombotic therapy was 16 weeks (2–104 weeks) (Table 2).

Thirteen patients (12.03%) underwent endovascular intervention. There were no reported complications for any patient who underwent endovascular intervention. Of these, 12 underwent endovascular intervention for management of documented dPSA. One patient did not have a documented dPSA and underwent endovascular management in the setting of worsening symptoms despite escalation to anticoagulation. dPSA size was reported in 7 of 12 patients who underwent endovascular therapy, with a mean size of 14.8 mm (2–20 mm). In contrast, among the 24 patients with dPSA who were managed without intervention, dPSA size was only reported in 6 cases, with a mean of 20.5 mm (6–30 mm). In the reported cases, the mean pseudoaneurysm size was 16.1 mm (2–26 mm) for cases involving the lower cranial nerves, 20 mm for the single case involving an upper cranial nerve, and 30 mm for the single case involving both upper and lower cranial nerves. No clear association was observed between pseudoaneurysm size and the level of cranial nerve involvement. The most common endovascular interventions included stenting (n = 4, 30.77%), followed by flow diverters (n = 3, 23.08%) and coil embolization (n = 3, 23.08%). Eight of the thirteen procedures were performed at the time of diagnosis, two were completed in the setting of clinical worsening, and two were completed due to interval changes seen on follow-up imaging. Of the four stents placed, three were performed in the setting of a diagnosed dissecting pseudoaneurysm (dPSA), and one was placed due to clinical worsening despite antithrombotic therapy. All three patients who underwent flow diversion had a diagnosed dPSA along with concerning clinical features, including progressive symptoms despite antithrombotic therapy and baroreflex failure. Among the three patients treated with coil embolization, two underwent the procedure at the time of initial dPSA diagnosis, while one had a delayed intervention due to persistent dPSA on follow-up imaging despite medical management.

### 3.5. Follow-Up and Outcomes

Outcomes were variably reported across studies, with some reporting only clinical data, others only imaging findings, and some reporting both. The follow-up duration ranged from 2 weeks to 154 weeks, with a mean of 21 weeks. Among patients who received only medical therapy, clinical outcomes were reported for 89 cases, of whom 84 (94.38%) showed complete or partial improvement. Of the cases that demonstrated complete to partial improvement, 41 patients (46.1%) received anticoagulant therapy, 32 patients (36.0%) received single antiplatelet therapy, and 11 patients (12.3%) received dual antiplatelet therapy. Imaging outcomes for these patients were available for 57 cases, with 49 (85.96%) demonstrating complete resolution or improvement of findings. In patients with dPSA, 20 out of 36 (55.5%) demonstrated partial and complete imaging improvement and 31 out of 36 (86.1%) demonstrated clinical improvement. In the group treated with endovascular procedures, imaging outcomes were available for nine patients, all of whom showed complete resolution without complications. Clinical outcomes were reported for 12 of these patients, all demonstrating either complete resolution or near-complete resolution of symptoms. No ischemic stroke or rupture of dissection/dPSA was observed in any of the 89 patients at follow-up.

## 4. Discussion

In this comprehensive literature review, we examined the baseline characteristics, clinical course, and clinical and radiographic outcomes of CAD-related cranial neuropathy in a predominantly young, male cohort. All reported patient cases of CAD-related cranial neuropathy occurred either spontaneously or secondarily to minor trauma. Lower cranial nerves were affected in the majority of cases, with fewer patients with associated upper cranial nerve palsies. Most patients were managed medically using antiplatelet or anticoagulant therapy; however, endovascular intervention was pursued in a small group of patients, especially in the setting of associated dPSA formation. Most patients demonstrated radiographic improvement or resolution, which was associated with improvement or resolution of their clinical symptoms. Overall, our findings underscore the excellent clinical and angiographic outcomes observed in CAD-related cranial neuropathy.

Consistent with prior studies, the patient population in our cohort was predominantly young and exhibited relatively few traditional vascular risk factors [4]. This aligns with the well-established observation that CAD often occurs in otherwise healthy individuals without significant atherosclerotic burden or conventional cardiovascular comorbidities. Although connective tissue disorders have been proposed as potential risk factors for CAD, evidence from previous cohort studies remains limited and inconclusive [107,108]. In our cohort, one patient had osteogenesis imperfecta. In contrast, CAD is a well-established manifestation of FMD, and three patients in our cohort had a diagnosis of FMD [109]. Most cases of CAD in the previous literature were reported to be spontaneous [3], and our findings support this trend. Notably, none of the patients in our cohort had a history of major trauma preceding the dissection.

Interestingly, we observed a higher proportion of male patients, with three fourths of cases occurring in men. This observation is consistent with findings from Arnold and colleagues, who reported that cranial neuropathy was twice as common in male patients with cervical artery dissection (CeAD) (8% vs. 4%) compared to females, although this difference did not reach statistical significance—which was likely due to limitations in sample size [110]. Notably, more recent data suggest that the overall incidence of CeAD is increasing among women [4]. This trend may reflect improved recognition of non-ischemic presentations, which appear to be more frequently seen in women [111]. Given this evolving understanding, it remains unclear whether the male predominance observed in our study reflects a true biological difference or if it is influenced by reporting bias. Further prospective and population-based studies are needed to better clarify the influence of sex on both the incidence and clinical presentation of CAD-related cranial neuropathy.

Lower cranial neuropathies are particularly susceptible to compression, given their close anatomical proximity to the skull base [8]. This can result either from an expansive intramural hematoma or a dPSA of the distal cervical ICA. Our findings support this hypothesis, as we observed cases of both isolated and combined lower cranial nerve palsies in the anatomical region adjacent to the distal cervical internal carotid artery. In contrast, upper cranial nerve involvement is more commonly attributed to ischemic phenomenon within steno-occlusive vertebral artery dissections but can also be secondary to microemboli or hypoperfusion of the vasa vasorum, particularly in dissections involving the more proximal cervical ICA segments [6]. Another possible ischemic mechanism for involvement of upper cranial neuropathies in CAD includes anatomical variability, such as the persistence of embryonic carotid–basilar anastomotic vessels, which may allow anterior circulation flow disruptions to affect regions that are typically supplied by the posterior circulation in conventional anatomical models. This mechanism has been specifically proposed in a case involving CN VII in combination with CN IX, X, and XII palsies secondary to CAD [7,10,11]. However, compressive neuropathy has also been proposed as an alternative explanation for the involvement of upper cranial nerves. One suggested mechanism involves direct compression at the cavernous segment of the internal carotid artery. Another posits that compression at the ostia of internal carotid artery branches, specifically the meningohypophyseal and inferolateral trunks, may secondarily affect the cranial nerves III, IV, V, and V through disruption of their vascular supply [10].

Although the association between CAD and arteriopathies is well established [108], our findings did not reveal a significant difference in the prevalence of known arteriopathies between patients with upper cranial nerve involvement and those with lower cranial nerve involvement. This suggests that these arteriopathies do not appear to be differentially associated with the pattern of cranial nerve palsies in CAD. In addition, we found that most cases across both lower and upper cranial nerve palsies occurred spontaneously and there was no clear association between pseudoaneurysm size and the extent or location of cranial nerve involvement. A prior cohort study demonstrated that peripheral cranial nerve palsies are frequently resolved within months, while brainstem ischemic-related palsies tend to persist over time [112]. This pattern was observed in our cohort, with patients demonstrating excellent clinical improvement and resolution on imaging within an average follow-up of five months.

CAD is an infrequent cause of stroke [113]. In our cohort, one patient initially presented with ischemic stroke, followed by the onset of lower cranial neuropathies. Although patients with CAD without ischemic stroke are considered to be at lower risk for thromboembolic events, antithrombotic therapy after CAD is typically recommended to prevent such complications [114,115]. However, the optimal antithrombotic regimen and its duration remain controversial. In our study, among the cases treated prior to the publication of the CADISS trial [114], anticoagulation was used more frequently than antiplatelet therapy. Following the publication of the CADISS trial, there was a noticeable shift toward antiplatelet use. Cases treated after 2017 predominantly used single antiplatelet therapy, with a marked decline in the use of anticoagulation. It remains to be seen whether this trend will shift again in light of emerging evidence from recent large-scale studies, such as the TREAT-CAD trial [116] and STOP-CAD study [5]. Despite clinical trials comparing antiplatelet and anticoagulant therapies, there remains no definitive evidence favoring one treatment over the other for CAD, including cases complicated by cranial nerve involvement. To date, these trials have shown no significant difference in stroke recurrence or clinical outcomes, underscoring the ongoing uncertainty regarding optimal medical management. It is also important to note that within the sub-cohort diagnosed with dPSA, there was an even distribution between dual and single antiplatelet therapy, whereas those without dPSA were more commonly managed with single antiplatelet therapy, suggesting potential differences in treatment approach based on lesion type. Regardless of the type or duration of antithrombotic used, most patients showed improvement or resolution of their symptoms, which is a biologically plausible finding, as the antithrombotic was not expected to have any effect on vascular remodeling or the consequent resolution of compression.

In our cohort, a subset of patients underwent endovascular intervention, the majority of whom were treated for diagnosed dPSA (either with or without clinical or radiographic worsening). One patient underwent intervention, despite the absence of a visible dPSA, due to worsening symptoms that persisted after escalated anticoagulation therapy. This case suggests that progressive clinical deterioration may justify intervention even in the absence of a detectable dPSA. Due to limited available data, there was no rationale provided on why one endovascular intervention was chosen over another. Interestingly, among patients with dPSA, those managed without endovascular intervention had a larger mean dPSA size compared to those managed with intervention, though data was limited. This overlap in size ranges underscores that factors beyond dPSA size, including symptom severity and risk profile, likely influenced treatment decisions. Consistent with current recommendations, endovascular stent placement was the most frequently used intervention [5]. Timing of intervention varied with eight procedures being performed at diagnosis based on clinical factors including baroreflex failure, high-risk thrombophilia, stroke prevention, and significant mass effect symptoms. Importantly, there were no reported complications, highlighting that this may be a safe option for escalation of care in a select cohort of patients. Together, these findings highlight the importance of individualized management strategies in which intervention is often driven by changing clinical and radiographic factors rather than size alone.

This study was subject to inherent limitations. Importantly, the retrospective nature of the study introduces the potential for reporting bias with a possible overrepresentation of patients who experienced more favorable clinical outcomes. The small sample size and heterogeneity of the data represent additional limitations, both of which are difficult to overcome given the rarity of this condition. In particular, the limited number of cases substantially restricts the statistical power and robustness of any observed trends, making it difficult to draw definitive conclusions. As a result, only descriptive analyses were feasible, and the findings should be interpreted with caution. In addition, detailed justification for the selection of specific treatment modalities, including stenting, coil embolization, or medical management, was limited, reflecting the limitations of relying on individual case reports and small case series. Lastly, the inclusion of only English-language articles may have resulted in the exclusion of relevant data from specific patient populations, leading to selection bias and limited generalizability.

## 5. Conclusions

Despite its limitations, this study represents the largest systematic evaluation of this rare clinical entity to our knowledge. Our findings suggest that CAD-related cranial neuropathy is generally a benign condition with a favorable recovery profile when treated conservatively. Endovascular therapy was only required in the few settings of dPSA or clinical worsening following conservative management. These results should be validated through larger prospective cohort studies, and further research is warranted to determine optimal management of CAD-associated cranial nerve palsies.

## Figures and Tables

**Table 1:** Baseline characteristics of 108 patients included in studies.

Variable	Number (%) or Mean (Range)
Age in years, mean (range)	48 (26–68)
Sex (female)	26 (24)
Cranial nerve involved	
CN III Isolated Combined	9 (8) 4 5
CN IV Isolated Combined	3 (3) 0 3
CN V Isolated Combined	8 (7) 6 2
CN VI Isolated Combined	5 (5) 1 4
CN VII Isolated Combined	7 (6) 4 3
CN VIII Isolated Combined	1 (1) 0 1
CN IX Isolated Combined	30 (28) 0 30
CN X Isolated Combined	38 (35) 3 35
CN XI Isolated Combined	21 (19) 1 20
CN XII Isolated Combined	82 (76) 45 37
**Pseudoaneurysms**	
**Yes**	36 (33)
**No**	72 (67)
ICA segment involved	
Cavernous	6 (6)
Petrous	21 (19)
Proximal cervical	7 (6)
Mid-cervical	23 (21)
Distal cervical	96 (89)
Etiology	
Minor trauma	22 (20)
Spontaneous	86 (80)

**Table 2:** Antithrombotic regimen and intervention details.

Variable	Number of Patients with Available Data	Number (%) or Mean (Range)
Antithrombotic regimen	100	
Anticoagulant Pre-CADISS Post-CADISS		43 (43) 39 4
Single antiplatelet Pre-CADISS Post-CADISS		37 (37) 20 15
Dual antiplatelet Pre-CADISS Post-CADISS		12 (12) 4 8
No medical treatment		10 (10)
Duration of antithrombotic therapy in weeks, mean (range)	80	16 (2–104) *
Interventional approach	13	
Stent		4 (30.77)
Flow diverter		3 (23.08)
Coil embolization		3 (23.08)
Stent coiling		2 (15.38)
Detachable balloon		1 (7.69)
Final follow-up time in weeks, mean (range)	98	21 (2–154) *

* Treatment and follow-up durations were converted to weeks; the last follow-up was used unless otherwise specified.

## Data Availability

Anonymized data are not published within this article will be made available upon request by any qualified investigator.

## Supplementary Materials

The following supporting information can be downloaded at: https://www.mdpi.com/article/10.3390/jcm14196854/s1, Supplementary Table S1: Database Search Strategies and Terms. Table S2: Baseline Characteristics, Antithrombotic Treatments, Interventions, and Reported Outcomes of Included Studies. Table S3: Joanna Briggs Institute Critical Appraisal Checklist for Case Reports.

## Abbreviations

The following abbreviations are used in this manuscript:

CADs	Carotid Artery Dissections
dPSA	Dissecting Pseudoaneurysms
CN XII	Hypoglossal Nerve
ICA	Internal Carotid Artery
RoB	Cochrane Risk of Bias
RCTs	Randomized Control Trials
NRSIs	Non-randomized studies of interventions
JBI	Joanna Brigg Institute
FMD	Fibromuscular Dysplasia
CN X	Vagus Nerve
CN XI	Glossopharyngeal Nerve
CN VII	Facial Nerve
CN VIII	Vestibulocochlear Nerve
CN V	Trigeminal Nerve
CN III	Oculomotor Nerve
CN IV	Trochlear Nerve
CN VI	Abducens Nerve
CeAD	Cervical Artery Dissection
