# Supplementary material for: Cranial Neuropathy Secondary to Carotid Artery Dissection: Clinical Features and Long-Term Outcomes
Source: J Clin Med. 2025 Sep 27;14(19):6854. doi: 10.3390/jcm14196854 (PMC12525036; doi:10.3390/jcm14196854)
Supplement: Supplementary file 1 [file jcm-14-06854-s001.zip › jcm-3820582-supplementary.pdf]

Supplementary Table S1. Database Search Strategies and Terms

Ovid

Database(s): EBM Reviews - Cochrane Central Register of Controlled Trials March 2025, Embase 1974 to 2025 April 14, Ovid MEDLINE(R) and Epub Ahead of Print, In-Process, In-Data-Review & Other Non-Indexed Citations, Daily and Versions 1946 to April 14, 2025
Search Strategy:

#	Searches	Results
1	exp Cranial Nerve Diseases/	318132
2	("Abducens Nerve Disease*" or "Abducens Nerve disorder*" or "Abducens Nerve palsies" or "Abducens Nerve palsy" or "Accessory Nerve Disease*" or "Accessory Nerve disorder*" or "Accessory Nerve palsies" or "Accessory Nerve palsy" or "cranial nerve disease*" or "cranial nerve disorder*" or "cranial nerve palsie*" or "cranial nerve palsies" or "cranial nerve palsy" or "cranial neuropathie*" or "cranial neuropathy" or "Facial Nerve Disease*" or "Facial Nerve disorder*" or "Facial Nerve palsies" or "Facial Nerve palsy" or "Glossopharyngeal Nerve Disease*" or "Glossopharyngeal Nerve disorder*" or "Glossopharyngeal Nerve palsies" or "Glossopharyngeal Nerve palsy" or "Hypoglossal Nerve Disease*" or "Hypoglossal Nerve disorder*" or "Hypoglossal Nerve palsies" or "Hypoglossal Nerve palsy" or "multiple cranial neuropathie*" or "multiple cranial neuropathy" or "nerve palsies" or "nerve palsy" or "nervus cranialis disorder*" or "Olfactory Nerve Disease*" or "Olfactory Nerve disorder*" or "Olfactory Nerve palsies" or "Olfactory Nerve palsy" or "Optic Nerve Disease*" or "Optic Nerve disorder*" or "Optic Nerve palsies" or "Optic Nerve palsy" or "Trochlear Nerve Disease*" or "Trochlear Nerve disorder*" or "Trochlear Nerve palsies" or "Trochlear Nerve palsy" or "Vagus Nerve Disease*" or "Vagus Nerve disorder*" or "Vagus Nerve palsies" or "Vagus Nerve palsy" or "Vestibulocochlear Nerve Disease*" or "Vestibulocochlear Nerve disorder*" or "Vestibulocochlear Nerve palsies" or "Vestibulocochlear Nerve palsy").ti,ab,kf.	48275
3	1 or 2	344351
4	exp Carotid Artery, Internal, Dissection/	7616
5	("carotid aneurysm" or "carotid artery aneurysm" or "carotid artery dissection" or "carotid artery pseudoaneurysm" or "carotid dissection" or "carotid pseudoaneurysm").ti,ab,kf.	9366
6	4 or 5	14463
7	3 and 6	1357
8	limit 7 to english language	1174
9	limit 7 to no language specified	4
10	8 or 9	1178
11	(exp animals/ or exp nonhuman/) not (exp humans/ or exp patient/)	12956822
12	((alpaca or alpacas or amphibian or amphibians or animal or animals or antelope or armadillo or armadillos or avian or baboon or baboons or beagle or beagles or bee or bees or bird or birds or bison or bovine or buffalo or buffaloes or buffalos or "c elegans" or "Caenorhabditis elegans" or camel or camels or canine or canines or carp or cats or cattle or chick or chicken or chickens or chicks or chimp or chimpanze or chimpanzees or chimps or cow or cows or "D melanogaster" or "dairy calf" or "dairy calves" or deer or dog or dogs or donkey or donkeys or drosophila or "Drosophila melanogaster" or duck or duckling or ducklings or ducks or equid or equids or equine or equines or feline or felines or ferret or ferrets or finch or finches or fish or flatworm or flatworms or fox or foxes or frog or frogs or "fruit flies" or "fruit fly" or "G mellonella" or "Galleria mellonella" or	10952842

	geese or gerbil or gerbils or goat or goats or goose or gorilla or gorillas or hamster or hamsters or hare or hares or heifer or heifers or horse or horses or insect or insects or jellyfish or kangaroo or kangaroos or kitten or kittens or lagomorph or lagomorphs or lamb or lambs or lemur or lemurs or llama or llamas or macaque or macaques or macaw or macaws or marmoset or marmosets or mice or minipig or minipigs or mink or minks or monkey or monkeys or mouse or mule or mules or nematode or nematodes or octopus or octopuses or orangutan or "orang-utan" or orangutans or "orang-utans" or ostrich or ostriches or oxen or parrot or parrots or pig or pigeon or pigeons or piglet or piglets or pigs or porcine or primate or primates or quail or rabbit or rabbits or rat or rats or reptile or reptiles or rodent or rodents or ruminant or ruminants or salmon or sheep or shrimp or slug or slugs or swine or tamarin or tamarins or toad or toads or trout or urchin or urchins or vole or voles or waxworm or waxworms or wildlife or worm or worms or xenopus or "zebra fish" or zebrafish) not (human or humans or patient or patients)).ti,ab,hw,kf.	
13	10 not (11 or 12)	1177
14	review.ti,pt.	7638155
15	13 not 14	994
16	(newborn* or neonat* or infant* or toddler* or child* or adolescent* or paediatric* or pediatric* or girl or girls or boy or boys or teen or teens or teenager* or preschooler* or "pre-schooler*" or preteen or preteens or "pre-teen" or "pre-teens" or youth or youths).ti,ab,hw,kf.	10859994
17	(adult or adulthood or adults or centenarian* or elderly or geriatric* or "middle age" or "middle aged" or nonagenarian* or octogenarian* or "old adult*" or "old people" or "old person*" or "older adult*" or "older people" or "older person*" or septuagenarian* or Sextenarian* or "very old").ti,ab,hw,kf.	21854345
18	16 not 17	6296471
19	13 not 18	1105
20	review.ti,pt. not ((case* adj3 report*) or retrospectiv* or chart).mp,pt.	6977259
21	19 not 20	1013
22	limit 21 to (conference abstract or conference paper or conference proceedings or preprint or pre-print or editorial or erratum or note or addresses or autobiography or bibliography or biography or blogs or comment or dictionary or directory or interactive tutorial or interview or lectures or legal cases or legislation or news or newspaper article or overall or patient education handout or periodical index or portraits or published erratum or video-audio media or webcasts or conference review or clinical trial protocol) [Limit not valid in CCTR,Embase,Ovid MEDLINE(R); records were retained]	180
23	21 not 22	833
24	remove duplicates from 23	646

Scopus

1 TITLE-ABS-KEY("Abducens Nerve Disease*" OR "Abducens Nerve disorder*" OR "Abducens Nerve palsies" OR "Abducens Nerve palsy" OR "Accessory Nerve Disease*" OR "Accessory Nerve disorder*" OR "Accessory Nerve palsies" OR "Accessory Nerve palsy" OR "cranial nerve disease*" OR "cranial nerve disorder*" OR "cranial nerve palsie*" OR "cranial nerve palsies" OR "cranial nerve palsy" OR "cranial neuropathie*" OR "cranial neuropathy" OR "Facial Nerve Disease*" OR "Facial Nerve disorder*" OR "Facial Nerve palsies" OR "Facial Nerve palsy" OR "Glossopharyngeal Nerve Disease*" OR "Glossopharyngeal Nerve disorder*" OR "Glossopharyngeal Nerve palsies" OR "Glossopharyngeal Nerve palsy" OR "Hypoglossal Nerve Disease*" OR "Hypoglossal Nerve disorder*" OR "Hypoglossal Nerve palsies" OR "Hypoglossal Nerve palsy" OR "multiple cranial neuropathie*" OR "multiple cranial neuropathy" OR "nerve palsies" OR "nerve palsy" OR "nervus cranialis disorder*" OR "Olfactory Nerve Disease*" OR "Olfactory Nerve disorder*" OR "Olfactory Nerve palsies" OR "Olfactory Nerve palsy" OR "Optic Nerve Disease*" OR "Optic Nerve disorder*" OR "Optic Nerve palsies" OR "Optic Nerve palsy" OR "Trochlear Nerve Disease*" OR "Trochlear Nerve disorder*" OR "Trochlear Nerve palsies" OR "Trochlear Nerve palsy" OR "Vagus Nerve Disease*" OR "Vagus Nerve disorder*" OR "Vagus Nerve palsies" OR "Vagus Nerve palsy" OR "Vestibulocochlear Nerve Disease*" OR "Vestibulocochlear Nerve disorder*" OR "Vestibulocochlear Nerve palsies" OR "Vestibulocochlear Nerve palsy")

2 TITLE-ABS-KEY("carotid aneurysm*" OR "carotid artery aneurysm*" OR "carotid artery dissection*" OR "carotid artery pseudoaneurysm*" OR "carotid dissection*" OR "carotid pseudoaneurysm*")

3 LANGUAGE(english)

4 1 and 2 and 3

5 TITLE-ABS-KEY((alpaca OR alpacas OR amphibian OR amphibians OR animal OR animals OR antelope OR armadillo OR armadillos OR avian OR baboon OR baboons OR beagle OR beagles OR bee OR bees OR bird OR birds OR bison OR bovine OR buffalo OR buffaloes OR buffalos OR "c elegans" OR "Caenorhabditis elegans" OR camel OR camels OR canine OR canines OR carp OR cats OR cattle OR chick OR chicken OR chickens OR chicks OR chimp OR chimpanze OR chimpanzees OR chimps OR cow OR cows OR "D melanogaster" OR "dairy calf" OR "dairy calves" OR deer OR dog OR dogs OR donkey OR donkeys OR drosophila OR "Drosophila melanogaster" OR duck OR duckling OR ducklings OR ducks OR equid OR equids OR equine OR equines OR feline OR felines OR ferret OR ferrets OR finch OR finches OR fish OR flatworm OR flatworms OR fox OR foxes OR frog OR frogs OR "fruit flies" OR "fruit fly" OR "G mellonella" OR "Galleria mellonella" OR geese OR gerbil OR gerbils OR goat OR goats OR goose OR gorilla OR gorillas OR hamster OR hamsters OR hare OR hares OR heifer OR heifers OR horse OR horses OR insect OR insects OR jellyfish OR kangaroo OR kangaroos OR kitten OR kittens OR lagomorph OR lagomorphs OR lamb OR lambs OR llama OR llamas OR macaque OR macaques OR macaw OR macaws OR marmoset OR marmosets OR mice OR minipig OR minipigs OR mink OR minks OR monkey OR monkeys OR mouse OR mule OR mules OR nematode OR nematodes OR octopus OR octopuses OR orangutan OR "orang-utan" OR orangutans OR "orang-utans" OR oxen OR parrot OR parrots OR pig OR pigeon OR pigeons OR piglet OR piglets OR pigs OR porcine OR primate OR primates OR quail OR rabbit OR rabbits OR rat OR rats OR reptile OR reptiles OR rodent OR rodents OR ruminant OR ruminants OR salmon OR sheep OR shrimp OR slug OR slugs OR swine OR tamarin OR tamarins OR toad OR toads OR trout OR urchin OR urchins OR vole OR voles OR waxworm OR waxworms OR worm OR worms OR xenopus OR "zebra fish" OR zebrafish) AND NOT (human OR humans or patient or patients))

6	4 and not 5
7	TITLE-ABS-KEY(newborn* or neonat* or infant* or toddler* or child* or adolescent* or paediatric* or pediatric* or girl or girls or boy or boys or teen or teens or teenager* or preschooler* or "pre-schooler*" or preteen or preteens or "pre-teen" or "pre-teens" or youth or youths) AND NOT TITLE-ABS-KEY(adult OR adulthood OR adults OR centenarian* OR elderly OR geriatric* OR "middle age" OR "middle aged" OR nonagenarian* OR octogenarian* OR "old adult*" OR "old people" OR "old person*" OR "older adult*" OR "older people" OR "older person*" OR septuagenarian* OR Sextenarian* OR "very old")
8	6 and not 7
9	TITLE(review)
10	TITLE-ABS-KEY((case* W/3 report*) or retrospectiv* or chart)
11	9 and not 10
12	8 and not 11
13	DOCTYPE(ab) OR DOCTYPE(ed) OR DOCTYPE(bk) OR DOCTYPE(er) OR DOCTYPE(no) OR DOCTYPE(sh)
14	12 and not 13
15	INDEX(embase) OR INDEX(medline) OR PMID(0* OR 1* OR 2* OR 3* OR 4* OR 5* OR 6* OR 7* OR 8* OR 9*)
16	14 and not 15

Supplemenary Table S2. Baseline Characteristics, Antithrombotic Treatments, Interventions, and Reported Outcomes *of Included Studies*

First Author, year	Age	Sex	Etiology	Cranial Nerve	Carotid Segment	Pseudoaneurysm	Medical Treatment	Intervention	Stent	Imaging Outcome	Clinical Outcome
Abuskeshek et al, 2022	56	Male	Spontaneous	12	Distal Cervical	Yes	None	Yes		Resolved	Improved
Ahmad et al, 2009	54	Male	Spontaneous	12	Distal Cervical	No	Single antiplatelet	No		Resolved	Not reported
Akova-Ozturk et al, 2004	65	Male	Spontaneous	9, 12	Distal Cervical	No	Anticoagulant	No		Resolved	Improved
Allingham et al, 2018	40	Male	Spontaneous	12	Distal Cervical	Yes	Single antiplatelet	No		Stable	Resolved
Arnolder et al, 2010	52	Male	Spontaneous	10,12	Distal Cervical	Yes	Anticoagulant	No	Flow diverter	Not reported	Improved
Athuraliya et al, 2017	64	Male	Trauma	9,12	Distal Cervical	Yes	Single antiplatelet	Yes		Not reported	Resolved
Bezerra et al, 2009	45	Male	Spontaneous	9,10,12	Distal Cervical, Petrosus	No	Single antiplatelet	No		Resolved	Improved
Bonkowsky et al, 2002	36	Male	Spontaneous	10,11,12	Mid Cervical, Distal Cervical	No	Anticoagulant	No		Resolved	Resolved
Boukobza et al,1998	38	Female	Spontaneous	12	Distal Cervical	No	Anticoagulant	No		Resolved	Resolved
Brajkovic et al, 2013	52	Male	Trauma	12	Distal Cervical	Yes	Single antiplatelet	No		Improved	Not reported
Campos et al, 2003	50	Male	Spontaneous	3	Mid Cervical	No	Anticoagulant	No		Not reported	Resolved
Caplan et al, 2012	35	Male	Spontaneous	9,10,11,12	Proximal Cervical, Mid Cervical, Distal Cervical	Yes	Anticoagulant	No		Improved	Resolved
Carancci et al, 2018	58	Male	Spontaneous	9,10,11,12	Distal Cervical	No	Single antiplatelet	No		Resolved	Resolved
Chen et al, 2019	42	Male	Spontaneous	12	Mid Cervical	No	Single antiplatelet	No	Coil embolization	Resolved	Resolved
Cruciata et al, 2017	56	Male	Spontaneous	12	Mid Cervical, Distal Cervical	Yes	Anticoagulant	Yes		Stable (before procedure)	Not reported
DeSantis et al, 2012	35	Female	Trauma	12	Mid Cervical, Distal Cervical	No	Anticoagulant	No		Resolved	Resolved
Dihne et al, 2000	37	Female	Spontaneous	3,4,6	Cavernous, Proximal Cervical, Mid Cervical, Distal Cervical	Yes	Anticoagulant	No		Resolved	Resolved
English et al, 2018	48	Male	Spontaneous	9,10,11,12	Distal Cervical	No	Not reported	No		Resolved	Not reported
Epinapov et, 2007	57	Male	Spontaneous	5	Petrosus	No	Anticoagulant	No		Resolved	Resolved
Epstein et al, 2012	45	Male	Spontaneous	12	Distal Cervical	No	Single antiplatelet	No	Flow diverter	Not reported	Improved
Erben et al, 2018	43	Female	Spontaneous	9,10,11,12	Distal Cervical	Yes	Dual antiplatelet	Yes	Coil emboliztation	Resolved	Resolved
Evan et al, 2021	42	Male	Spontaneous	9,10,11,12	Distal Cervical	Yes	Dual antiplatelet	Yes		Resolved	Resolved
Ferlazzo et al, 2013	37	Male	Spontaneous	12	Distal Cervical	No	Anticoagulant	No		Not reported	Improved
Fink et al, 1998	53	Male	Spontaneous	10,11,12	Mid Cervical	No	Single antiplatelet	No		Improved	Improved
Freilinger et al, 2010	35	Male	Trauma	12	Distal Cervical	No	Anticoagulant	No		Not reported	Resolved
Fujii et al, 2014	42	Male	Trauma	12	Distal Cervical	Yes	Not reported	No		Improved	Resolved
Graham et al, 2014	68	Female	Spontaneous	12	Distal Cervical	Yes	Single antiplatelet	No		Improved	Improved
Guidetti et al, 2001	49	Female	Spontaneous	11,12	Distal Cervical, Petrosus	No	Anticoagulant	No		Resolved	Resolved

Supplemenary Table S2. Baseline Characteristics, Antithrombotic Treatments, Interventions, and Reported Outcomes *of Included Studies*

Guidetti et al, 2001	38	Female	Spontaneous	11	Mid Cervical, Distal Cervical	No	Anticoagulant	No		Resolved	Improved
Guy et al, 2001	60	Male	Spontaneous	12	Petrosus	No	Anticoagulant	No		Improved	Resolved
Guy et al, 2001	49	Male	Spontaneous	9,10,12	Mid Cervical, Distal Cervical, Petrousus	No	Anticoagulant	No		Not reported	Resolved
Guy et al, 2001	51	Male	Trauma	12	Distal Cervical	No	Anticoagulant	No		Resolved	Resolved
Hafkamp et al, 2004	61	Male	Spontaneous	12	Distal Cervical	No	None	No		Not reported	Improved
Hegde et al, 2002	60	Female	Trauma	3	Petrosus	No	Not reported	No		Not reported	Resolved
Hennings et al, 2014	49	Female	Trauma	12	Distal Cervical	Yes	Anticoagulant	No		Improved	Resolved
Herath et al, 2017	38	Female	Spontaneous	3,4,6	Cavernous	Yes	Single antiplatelet	No		Stable	Stable
Herath et al, 2024	54	Male	Trauma	7	Distal Cervical	No	Single antiplatelet	No		Not reported	Resolved
Hess et al, 1990	41	Male	Spontaneous	10,12	Distal Cervical	No	Anticoagulant	No		Stable	Improved
Himstead et al, 2023	45	Female	Spontaneous	6	Petrosus	Yes	Single antiplatelet	No	Stent coiling	Resolved	Improved
Im et al, 2020	26	Female	Spontaneous	3,4,6	Cavernous	Yes	Dual antiplatelet	Yes	Stent	Not reported	Improved
Introna et al, 2017	37	Female	Spontaneous	10,12	Distal Cervical	Yes	Dual antiplatelet	Yes	Stent	Resolved	Resolved
Jaume et al, 2015	29	Male	Trauma	9,10,12	Distal Cervical	Yes	Dual antiplatelet	Yes		Not reported	Improved
Jurkiewicz et al, 2019	52	Female	Spontaneous	12	Distal Cervical	No	Anticoagulant	No		Not reported	Improved
Jurkiewicz et al, 2019	46	Male	Spontaneous	12	Distal Cervical	No	Dual antiplatelet	No		Not reported	Resolved
Jurkiewicz et al, 2019	38	Male	Spontaneous	12	Proximal Cervical, Mid Cervical, Distal Cervical, Petrosus	No	Single antiplatelet	No		Not reported	Improved
Jurkiewicz et al, 2019	47	Male	Trauma	12	Distal Cervical	No	Single antiplatelet	No		Not reported	Stable
Kasrawi et al, 2010	61	Male	Trauma	12	Distal Cervical, Petrosus	No	Single antiplatelet	No		Resolved	Improved
Kaushik et al, 2009	44	Male	Spontaneous	12	Distal Cervical, Petrous	Yes	Anticoagulant	No	Stent	Not reported	Resolved
Kidoguchi et al, 2022	47	Male	Spontaneous	12	Distal Cervical	No	Dual antiplatelet	Yes		Resolved	Improved
Klossek et al, 1994	49	Male	Spontaneous	9,10,11,12	Distal Cervical	No	Anticoagulant	No		Resolved	Not reported
Knibb et al, 2005	47	Male	Spontaneous	10,12	Mid Cervical	No	Single antiplatelet	No		Stable	Resolved
Kondo et al, 2022	52	Male	Spontaneous	7	Distal Cervical	No	None	No		Not reported	Improved
Liescchke et al, 1988	42	Male	Spontaneous	12	Distal Cervical	Yes	None	No		Stable	Resolved
Lindsay et al, 2003	43	Male	Spontaneous	12	Distal Cervical	No	Anticoagulant	No	Coil embolization	Resolved	Resolved
Lin et al, 2023	49	Female	Spontaneous	9,10,11,12	Distal Cervical	Yes	None	Yes		Resolved	Improved
Lombardi et al, 2022	51	Male	Spontaneous	12	Distal Cervical	No	Single antiplatelet	No		Not reported	Resolved
Majeed et al, 2016	55	Male	Spontaneous	7,10,12	Distal Cervical	Yes	Anticoagulant	No		Not reported	Resolved

Supplemenary Table S2. Baseline Characteristics, Antithrombotic Treatments, Interventions, and Reported Outcomes *of Included Studies*

Makhlouf et al, 2013	60	Female	Trauma	7	Distal Cervical	Yes	Anticoagulant	No		Not reported	Resolved
Marin et al, 2009	52	Male	Spontaneous	12	Distal Cervical, Cavernous	Yes	Anticoagulant	No		Not reported	Improved
Mattioni et al, 2007	66	Male	Spontaneous	8,9,10,12	Proximal Cervical, Mid Cervical, Distal Cervical	No	Single antiplatelet	No		Resolved	Resolved
McCarron et al, 2000	44	Male	Spontaneous	7	Proximal Cervical	No	Anticoagulant	No		Resolved	Resolved
Moussouttas et al,1998	40	Male	Spontaneous	10	Distal Cervical	No	Not reported	No		Not reported	Not reported
Murnane et al,1996	62	Male	Spontaneous	5	Proximal Cervical, Mid Cervical, Distal Cervical, Petrous, Cavernous	No	Anticoagulant	No		Not reported	Resolved
Nakagawa et al, 2014	57	Male	Spontaneous	10	Distal Cervical	No	None	No		Resolved	Resolved
Neau et al, 1993	49	Male	Spontaneous	9,10,11,12	Distal Cervical, Petrous	Yes	Anticoagulant	No		Resolved	Resolved
Nowak et al, 2022	40	Male	Spontaneous	9,10,11,12	Mid Cervical, Distal Cervical	No	None	No		Resolved	Improved
Okunomiya et al, 2012	57	Male	Spontaneous	12	Distal Cervical	No	Single antiplatelet	No		Not reported	Resolved
Pakdemirli et al, 2001	52	Male	Spontaneous	12	Mid Cervical, Distal Cervical	No	Anticoagulant	No		Resolved	Resolved
Panisset et al,1990	36	Male	Spontaneous	9,10,11,12	Distal Cervical, Petrous	No	None	No		Resolved	Resolved
Panisset et al,1990	53	Male	Trauma	5,7,9,10,12	Distal Cervical	No	None	No		Resolved	Resolved
Pawlukowska et al, 2023	36	Female	Trauma	9,10,12	Distal Cervical	Yes	Dual antiplatelet	No		Improved	Improved
Peltz et al, 2011	45	Female	Spontaneous	9,10,12	Distal Cervical	No	None	No		Not reported	Resolved
Pica et al, 1996	31	Female	Trauma	12	Distal Cervical	Yes	Anticoagulant	No		Resolved	Improved
Pikjia et al, 2014	66	Male	Spontaneous	12	Distal Cervical	No	Anticoagulant	No		Not reported	Improved
Pongmoragot et al, 2013	60	Male	Trauma	12	Distal Cervical	No	Single antiplatelet	No	Stent coiling	Not reported	Resolved
Popov et al, 2016	44	Female	Trauma	10	Proximal Cervical, Distal Cervical	Yes	Anticoagulant	Yes		Resolved	Resolved
Qi et al, 2015	45	Female	Spontaneous	12	Distal Cervical	Yes	Dual antiplatelet	No		Not reported	Resolved
Rianco et al, 2013	54	Male	Spontaneous	12	Distal Cervical	No	Dual antiplatelet	No		Not reported	Improved
Ryan et al, 2015	52	Male	Spontaneous	12	Distal Cervical	No	Single antiplatelet	No		Not reported	Resolved
Saliou et al, 2018	61	Male	Spontaneous	7,9,10,11,12	Distal Cervical	Yes	Methylprednisolone	No		Resolved	Resolved
Santos et al, 2014	58	Male	Spontaneous	3	Distal Cervical, Petrous	No	Single antiplatelet	No		Resolved	Resolved
Sasaki et al, 2018	39	Male	Spontaneous	9,10	Distal Cervical	No	Single antiplatelet	No		Resolved	Improved
Schievik et al, 1993	35	Female	Spontaneous	3,5	Distal Cervical, Petrous	No	Not reported	No		Not reported	Improved
Selky et al,1995	38	Female	Spontaneous	5	Distal Cervical, Petrous	No	Single antiplatelet	No		Resolved	Improved
Shabab et al, 2001	46	Male	Spontaneous	12	Distal Cervical	Yes	Single antiplatelet	No		Not reported	Stable
Shi et al, 2022	50	Male	Trauma	9,10,11,12	Distal Cervical	No	Dual antiplatelet	No		Resolved	Resolved

Supplemenary Table S2. Baseline Characteristics, Antithrombotic Treatments, Interventions, and Reported Outcomes *of Included Studies*

Simionescu et al, 2004	64	Male	Trauma	10,12	Proximal Cervical, Mid Cervical, Distal Cervical, Petrous	No	Single antiplatelet	No		Not reported	Resolved
Simpson et al, 2021	43	Male	Spontaneous	12	Distal Cervical	No	Single antiplatelet	No		Not reported	Resolved
Smith et al, 2013	52	Male	Spontaneous	9,10,11,12	Distal Cervical	No	Single antiplatelet	No		Not reported	Improved
Spitzer et al, 2001	39	Male	Spontaneous	12	Distal Cervical, Petrous	No	Anticoagulant	No		Resolved	Resolved
Srinivas et al, 2021	53	Male	Spontaneous	12	Distal Cervical	No	Single antiplatelet	No		Not reported	Improved
Stefani et al, 1996	39	Male	Spontaneous	5	Distal Cervical	No	Single antiplatelet	No		Worsened (new disection contralateral ICA)	Stable
Stubgen et al, 2011	56	Male	Spontaneous	12	Distal Cervical	Yes	Single antiplatelet	No		Not reported	Resolved
Sturzenegger et al, 1993	42	Male	Trauma	9,10	Distal Cervical, Petrous	No	Anticoagulant	No		Resolved	Resolved
Sturzenegger et al, 1993	45	Male	Spontaneous	12	Distal Cervical	Yes	Anticoagulant	No		Improved	Stable
Theodorou et al, 2024	68	Male	Spontaneous	9,10,11,12	Distal Cervical	No	Single antiplatelet	No		Not reported	Improved
Torbus-Paluszczak et al, 2018	50	Male	Spontaneous	12	Mid Cervical, Distal Cervical	No	Anticoagulant	No		Stable	Improved
Torbus-Paluszczak et al, 2018	48	Male	Spontaneous	9,12	Distal Cervical	No	Anticoagulant	No		Resolved	Improved
Ursekar et al, 2000	37	Female	Spontaneous	12	Mid Cervical, Distal Cervical, Petrousus	Yes	Anticoagulant	No		Not reported	Improved
van der Zwet et al, 2020	54	Female	Spontaneous	5	Mid Cervical, Distal Cervical, Petrous	No	Single antiplatelet	No		Not reported	Resolved
Verdalle et al, 2001	65	Male	Spontaneous	12	Distal Cervical	No	Anticoagulant	No		Not reported	Resolved
Verdalle et al, 2001	54	Male	Spontaneous	9,10,12	Mid Cervical, Distal Cervical	No	Anticoagulant	No	Detachable balloon	Improved	Resolved
Waespe et al, 1988	41	Male	Trauma	9,10,11,12	Distal Cervical	Yes	None	Yes		Improved	Improved
Walker et al, 2003	54	Male	Spontaneous	9,10,11,12	Distal Cervical	No	Single antiplatelet	No		Resolved	Improved
Wessels et al, 2005	55	Male	Spontaneous	3	Without Cavernous	No	Anticoagulant	No		Improved	Improved
Wessels et al, 2005	45	Male	Spontaneous	5	Without Cavernous	No	Anticoagulant	No	Flow diverter	Resolved	Not reported
Zelenak et al, 2013	46	Male	Spontaneous	9,10,11,12	Distal Cervical	Yes	Dual antiplatelet	Yes		Resolved	Resolved
Zhou et al, 2010	49	Female	Spontaneous	3,6	Cavernous	No	None	No		Resolved	Resolved

Supplementary Table S3. Joanna Briggs Institute Critical Appraisal Checklist for Case Reports

First Author, year	Demographics Described	Patient history presented as timeline	Description of current clinical condition	Description of diagnostic tests and results	Description of intervention/ treatment	Description of postintervention condition	Description of adverse events	Take away lessons	Risk of Bias
Abuskeshek et al, 2022	Y	Y	Y	Y	Y	Y	Y	Y	Low
Ahmad et al, 2009	Y	Y	Y	Y	Y	Y	Y	Y	Low
Akova-Ozturk et al, 2004	Y	Y	Y	Y	Y	Y	Y	Y	Low
Allingham et al, 2018	Y	Y	Y	Y	Y	Y	Y	Y	Low
Arnolder et al, 2010	Y	Y	Y	Y	Y	U	Y	Y	Low
Athuraliya et al, 2017	Y	Y	Y	Y	Y	U	Y	Y	Low
Bezerra et al, 2009	Y	Y	Y	Y	Y	Y	Y	Y	Low
Bonkowsky et al, 2002	Y	Y	Y	Y	Y	Y	Y	Y	Low
Boukobza et al, 1998	Y	Y	Y	Y	Y	Y	Y	Y	Low
Brajkovic et al, 2013	Y	Y	Y	Y	Y	U	Y	Y	Low
Campos et al, 2003	Y	Y	Y	Y	Y	U	Y	Y	Low
Caplan et al, 2012	Y	Y	Y	Y	Y	Y	Y	Y	Low
Caranci et al, 2018	Y	Y	Y	Y	Y	Y	Y	Y	Low
Chen et al, 2019	Y	Y	Y	Y	Y	Y	Y	Y	Low
Cruciata et al, 2017	Y	Y	Y	Y	Y	N	Y	Y	Low
DeSantis et al, 2012	Y	Y	Y	Y	Y	Y	Y	Y	Low
Dihne et al, 2000	Y	Y	Y	Y	U	U	U	Y	Medium
English et al, 2018	Y	Y	Y	Y	N	U	N	Y	Medium
Epinapov et al, 2007	Y	Y	Y	Y	Y	Y	Y	Y	Low
Epstein et al, 2012	Y	Y	Y	Y	Y	U	Y	Y	Low
Erben et al, 2018	Y	Y	Y	Y	Y	Y	Y	Y	Low
Evan et al, 2021	Y	Y	Y	Y	Y	Y	Y	Y	Low
Ferlazzo et al, 2013	Y	Y	Y	Y	Y	U	Y	Y	Low
Fink et al, 1998	Y	Y	Y	Y	Y	Y	Y	Y	Low
Freilinger et al, 2010	Y	Y	Y	Y	Y	U	Y	Y	Low

Fujii et al, 2014	Y	Y	Y	Y	N	U	N	Y	Medium
Graham et al, 2014	Y	Y	Y	Y	Y	Y	Y	Y	Low
Guidetti et al, 2001	Y	Y	Y	Y	Y	Y	Y	Y	Low
Guy et al, 2001	Y	Y	Y	Y	Y	Y	Y	Y	Low
Hafkamp et al, 2004	Y	Y	Y	Y	N	U	N	Y	Medium
Hedge et al, 2002	Y	Y	Y	Y	N	U	N	Y	Medium
Hennings et al, 2014	Y	Y	Y	Y	Y	Y	Y	Y	Low
Herath et al, 2017	Y	Y	Y	Y	Y	Y	Y	Y	Low
Herath et al, 2024	Y	Y	Y	Y	Y	Y	Y	Y	Low
Hess et al, 1990	Y	Y	Y	Y	Y	Y	Y	Y	Low
Himstead et al. 2023	Y	Y	Y	Y	Y	Y	Y	Y	Low
Im et al, 2020	Y	Y	Y	Y	Y	U	Y	Y	Low
Introna et al, 2017	Y	Y	Y	Y	Y	Y	Y	Y	Low
Jaume et al, 2015	Y	Y	Y	Y	Y	U	Y	Y	Low
Jurkiewicz et al, 2019	Y	U	Y	Y	Y	U	Y	Y	Low
Kasrawi et al, 2010	Y	Y	Y	Y	Y	Y	Y	Y	Low
Kaushik et al, 2009	Y	Y	Y	Y	Y	U	Y	Y	Low
Kidoguchi et al, 2022	Y	Y	Y	Y	Y	Y	Y	Y	Low
Klossek et al, 1994	Y	Y	Y	Y	Y	Y	Y	Y	Low
Knibb et al, 2005	Y	Y	Y	Y	Y	Y	Y	Y	Low
Kondo et al, 2022	Y	Y	Y	Y	N	U	N	Y	Medium
Liescchke et al, 1988	Y	Y	Y	Y	N	Y	N	Y	Medium
Lindsay et al, 2003	Y	Y	Y	Y	Y	Y	Y	Y	Low
Lin et al, 2003	N	N	Y	Y	Y	Y	Y	N	Medium
Lombardi et al, 2022	Y	Y	Y	Y	Y	U	Y	Y	Low
Majeed et al, 2016	Y	Y	Y	Y	Y	U	Y	Y	Low
Makhlouf et al, 2013	Y	Y	Y	Y	Y	U	Y	Y	Low
Marin et al, 2009	Y	Y	Y	Y	Y	U	Y	Y	Low
Mattioni et al, 2007	Y	Y	Y	Y	Y	Y	Y	Y	Low
McCarron et al, 2000	Y	Y	Y	Y	Y	Y	Y	Y	Low

Moussouttas et al, 1998	Y	N	Y	Y	N	N	N	Y	High
Murnane et al, 1996	Y	Y	Y	Y	Y	U	Y	Y	Low
Nakagawa et al, 2014	Y	Y	Y	Y	Y	Y	Y	Y	Low
Neau et al, 1993	Y	Y	Y	Y	Y	Y	Y	U	Low
Nowak et al, 2022	Y	Y	Y	Y	Y	Y	Y	Y	Low
Okunomiya et al, 2012	N	N	Y	Y	Y	U	Y	Y	Medium
Pakdemirli et al, 2001	Y	Y	Y	Y	Y	Y	Y	Y	Low
Panisset et al, 1990	Y	Y	Y	Y	N	Y	N	Y	Low
Pawlukowska et al, 2023	Y	Y	Y	Y	Y	Y	Y	Y	Low
Peltz et al, 2011	Y	Y	Y	Y	N	U	N	Y	Medium
Pica et al, 1996	Y	Y	Y	Y	Y	Y	Y	Y	Low
Pikjia et al, 2014	Y	Y	Y	Y	Y	U	Y	U	Low
Pongmoragot et al, 2013	Y	Y	Y	Y	Y	U	Y	Y	Low
Popov et al, 2016	Y	Y	Y	Y	Y	Y	Y	Y	Low
Qi et al, 2015	Y	Y	Y	Y	Y	U	Y	Y	Low
Rianco et al, 2013	Y	Y	Y	Y	Y	U	Y	Y	Low
Ryan et al, 2015	Y	Y	Y	Y	Y	U	Y	Y	Low
Saliou et al, 2018	Y	Y	Y	Y	Y	Y	Y	Y	Low
Santos et al, 2014	Y	Y	Y	Y	Y	Y	Y	Y	Low
Sasaki et al, 2018	Y	Y	Y	Y	Y	Y	Y	Y	Low
Schievik et al, 1993	Y	Y	Y	Y	U	U	Y	Y	Low
Selky et al, 1995	Y	Y	Y	Y	Y	Y	Y	Y	Low
Shabab et al, 2001	Y	Y	Y	Y	Y	U	Y	Y	Low
Shi et al, 2022	Y	Y	Y	Y	Y	Y	Y	Y	Low
Simionescu et al, 2004	Y	Y	Y	Y	Y	U	Y	Y	Low
Simpson et al, 2021	Y	Y	Y	Y	Y	U	Y	Y	Low
Smith et al, 2013	Y	Y	Y	Y	Y	U	Y	Y	Low
Spitzer et al, 2001	Y	Y	Y	Y	Y	Y	Y	Y	Low

Srinivas et al, 2021	Y	Y	Y	Y	Y	U	Y	Y	Low
Stefani et al, 1996	Y	Y	Y	Y	Y	Y	Y	Y	Low
Stugben et al, 2011	Y	Y	Y	Y	Y	U	Y	Y	Low
Sturzenegger et al, 1993	Y	Y	Y	Y	Y	Y	Y	Y	Low
Theodorou et al, 2024	Y	Y	Y	Y	Y	U	Y	Y	Low
Torbus-Paluszczak et al, 2018	Y	Y	Y	Y	Y	Y	Y	Y	Low
Ursekar et al, 2000	Y	Y	Y	Y	Y	U	Y	Y	Low
van der Zwet et al, 2020	Y	Y	Y	Y	Y	U	Y	Y	Low
Verdalle et al, 2001	Y	Y	Y	Y	Y	Y	Y	Y	Low
Waespe et al, 1988	Y	Y	Y	Y	Y	Y	Y	Y	Low
Walker et al, 2003	Y	Y	Y	Y	Y	Y	Y	Y	Low
Wessels et al, 2005	Y	Y	Y	Y	Y	Y	Y	Y	Low
Zelenak et al, 2013	Y	Y	Y	Y	Y	Y	Y	Y	Low
Zhou et al, 2010	Y	Y	Y	Y	Y	Y	Y	Y	Low

Y = Yes; N = No; U = Unclear
